# Artificial Intelligence Assisted Ultrasonic Extraction of Total Flavonoids from *Rosa sterilis*

**DOI:** 10.3390/molecules26133835

**Published:** 2021-06-23

**Authors:** Jing Liu, Chaochan Li, Guijie Ding, Wenxuan Quan

**Affiliations:** 1 Key Laboratory of Plant Physiology and Developmental Regulation of Guizhou Province, College of Life Sciences, Guizhou Normal University, Guiyang 550025, China; 19010100278@gznu.edu.cn; 2 Guizhou Provincial Key Laboratory for Information Systems of Mountainous Areas and Protection of Ecological Environment, Guizhou Normal University, Guiyang 550001, China; wenxuanq@gznu.edu.cn; 3 Key Laboratory of Forest Cultivation in Plateau Mountain of Guizhou Province, Institute for Forest Resources and Environment of Guizhou, Guizhou University, Guiyang 550025, China

**Keywords:** *Rosa sterilis*, ultrasonic, artificial intelligence, extraction, free radical scavenging, kinetics

## Abstract

Flavonoids in *Rosa sterilis* were studied. The flavonoids in *Rosa sterilis* were extracted by ultrasonic method, and the extraction conditions were modeled and optimized by response the surface methodology and the artificial intelligence method. The results show that the ultrasonic method can effectively extract total flavonoids, and the extraction rate is close to the prediction value of ANN-GA algorithm, which proves the rationality of the model. The order of the effects of the parameters on the experiment was material liquid ratio > extraction power > extraction time > ethanol concentration. In addition, the scavenging effects of flavonoids on DPPH, O^2−^· and ·OH were also determined, and these indicated that flavonoids have strong antioxidant activities. The kinetics of the extraction process was studied by using the data of the extraction process, and it was found that the extraction process conformed to Fick’s first law.

## 1. Introduction

**Figure 1:**
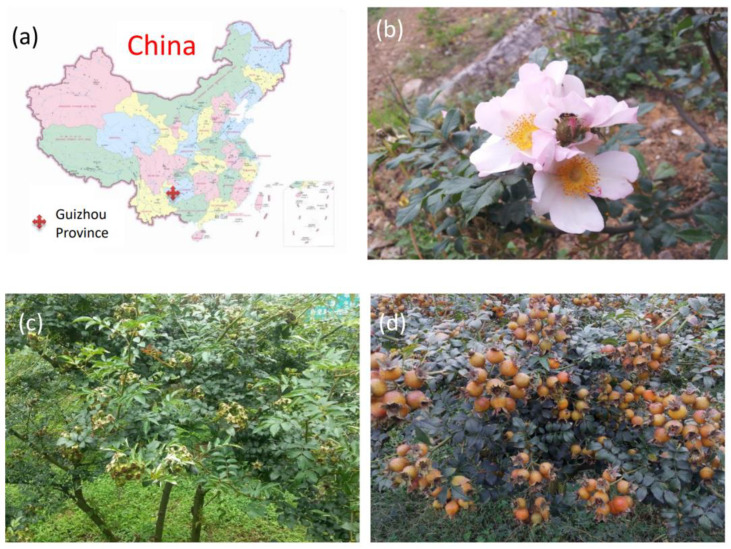
Origin and growth habit of *Rosa sterilis*. (**a**) the place of origin is Guizhou Province; (**b**) flowers (May); (**c**) green fruits (July); (**d**) mature fruits (October).

*Rosa roxburghii* Tratt belongs to Rosaceae, one of the important economic trees, and is planted widely in the southwest and central south mountainous areas of China [1]. It is rich in vitamin C, protein, polysaccharide, amino acids and other active substances. *Rosa roxburghii* has good ornamental, edible (dried fruits and drinks) and medicinal value (anti-aging, anti-mutation, anti-tumor, ovarian cancer cell metastasis, atherosclerosis and hyperlipidemia, etc.). *Rosa sterilis* S.D. Shi, is a relative of *Rosa roxburghii*. This plant species was discovered in 1983 and is propagated through cutting and promotion; it is an endemic plant resource in Guizhou Province (Figure 1). The contents of total sugar, flavone, superoxide dismutase and vitamin in the fruit of *Rosa sterilis* are higher than those in *Rosa roxburghii*, and the taste is also better; the fruit is sweet and slightly astringent. The average content of flavonoids is 680 mg/100 g in *Rosa roxburghi*, and the highest content was 800 mg/100g [2,3,4,5].

**Figure 2:**
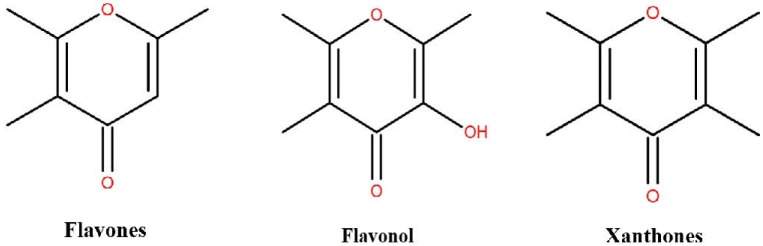

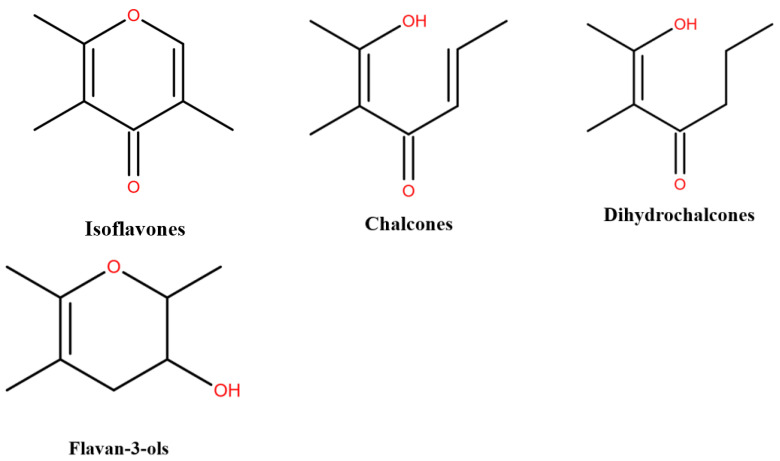
Structure of different flavonoids.

Flavonoids are the secondary metabolites of plants. In the natural environment, they appear in the form of simple molecules [6]. Flavonoids are divided into chalcone, flavonoids, flavonols, dihydroflavonols, anthocyanins and proanthocyanidins (Figure 2). They play a key role in anti-oxidation [7,8] and are anti-inflammatory [9,10], anti-virus [11,12], antibacterial [13,14], anti-tumor [15,16], UV resistant [17], and plant growth and reproduction [18,19]. They are important substances in drugs and personal care products. Flavonoids can effectively scavenge free radicals as antioxidants, thus maintaining the balance of oxidation and antioxidation in the body and eliminating the damage to the biological system of the radicals [20].

There are many methods for extracting flavonoids at home and abroad, e.g., organic solvent extraction [21], enzymatic hydrolysis [22], resin adsorption [23,24], high performance liquid chromatography [25,26], supercritical extraction [27], microwave extraction [28,29], ultrasonic extraction technology [30,31], and soxhlet extraction [32]. Ultrasonic extraction technology is a new method for extracting active ingredients from plants in recent years and which works by using the strong vibration, cavitation and comminution generated by ultrasonic to extract the active ingredients from plants into solvent. The method is simple, efficient, and less by-products, and it can achieve better results than conventional extraction. The by-products produced in the process of extracting flavonoids include protein, fiber, starch, polysaccharide compounds, and flavonoids derivatives [33,34].

Response Surface Methodology (RSM) refers to obtaining a constant data by experiments using a rational experimental design method. Its advantage is to save time and reduce the consumption of reagents and materials. Artificial intelligence (AI) has good nonlinear mapping, generalization, self-organization and self-learning capabilities, which have been widely used in robots, language recognition, image recognition, aerospace application, and automatic operations [35,36,37]. Genetic algorithm (GA) and particle swarm optimization (PSO) are used to model and globally optimize the flavonoid extraction process. F-test, random forest (RF), radial basis function (RBF), and gradient boosting regression tree (GBRT) were used to search and sort the influencing factors of flavonoids extraction, which is beneficial to improve the extraction efficiency of flavonoids.

The main purpose of this study was to study the ultrasonic assisted extraction of flavonoids from the mature fruits of *Rosa sterilis*. Artificial intelligence tools (ANN-GA, ANN-PSO, RF and RBF) were combined with response surface methodology (RSM) to optimize the extraction efficiency of flavonoids in *Rosa sterilis*. The effects of extraction time, ethanol concentration, material liquid ratio, and extraction power on the extraction efficiency of flavonoids were investigated through batch experiments. Garson formula, RF, F-test, and GBRT were used to evaluate the importance of the four factors in the extraction process. The results showed that material liquid ratio had the greatest influence on the extraction of flavonoids. In addition, the extraction kinetics of flavonoids was studied.

## 2. Materials and Methods

### 2.1. Materials

Mature fruits of Rosa sterilis from Liupanshui City, Guizhou Province, China, were used. They were washed with distilled water, and the fruit with full grains and no mold was selected and stored at 4 °C for reserve. The frozen *Rosa sterilis* was not dry and damaged.

All of the chemical reagents used in this paper were of analytical grade and were used without further purification.

### 2.2. Instruments and Equipment

UV756 ultraviolet visible spectrophotometer (Shanghai Yuanxi Instrument Co., Shanghai, China), WH-600 Ultrasonic Cleaner (Jining Wanhe Ultrasonic Electronic Equipment Co., Shandong, China), BSM Electronic Balance (Shanghai Zhuojing Electronic Technology Co., Shanghai, China), SC-3610 low-speed centrifuge (Anhui Zhongke Zhongjia Scientific Instrument Co., Anhui, China).

The instrument parameters of this ultraviolet spectrophotometer are as follows: spectral measurement wavelength range is 190–1100 nm, wavelength accuracy is ±5 nm, wavelength repeatability is 0.2 nm, spectral width is 2 nm, transmittance accuracy is 0 ± 5% T, transmission specific gravity refolding is 0.2% T, luminosity range is—3~3a, 0–200% T, 0~9999 C, baseline straightness is ±001 A/h, stray light is 0.05% T@220 nm and 360 nm, Stability is ±001 a/h @ 500 nm.

## 3. Experimental Methods

### 3.1. Single Factor Experiment

#### 3.1.1. Making of Standard Curve

NaNO_2_-Al (NO_3_) _3_-NaOH colorimetric method was used in this experiment [38]. The product of 10 mg rutin standard was accurately measured, put into a 50 mL volumetric flask, and the volume was fixed with 60% ethanol to obtain a rutin standard concentration solution of 0.2 mg/mL. In addition, each of the precise amounts is 0.00 mL, 1.00 mL, 2.00 mL, 3.00 mL, 4.00 mL and 5.00 mL of rutin standard solution were accurately measured and placed in six 10 mL volumetric flasks, respectively. Ethanol with a concentration of 60% was added to 5 mL and 5% NaNO_2_ to 0.3 mL respectively, and then the solution was allowed to stand for 5 min. Next, 10% Al (NO_3_)_3_ (0.3 mL) was added to the above solution and allowed to stand for 6 min. Then, 4% NaOH (2 mL) was added, 60% ethanol was used to fix the volume to 10 mL, and shaken well and allowed to stand for 10 min to obtain the supernatant with a pipette. The absorbance of the supernatant was measured at 507 nm respectively, the concentration of rutin was abscissa, and the absorbance was ordinate. The standard curve equation was Y = 5.0629x + 0.1885, and R^2^ was 0.9996.

The flavonoids efficiency could be calculated by Formula (1):

(1) W%=(C×V)/m×100%

where *W*% was flavonoids efficiency; *C* was the mass concentration of flavonoids in the sample (g/mL); *V* was the total volume of the extract (mL); and m was the mass of *Rosa sterilis* (g).

#### 3.1.2. Single Factor Experiment

Single factor experiments were conducted to investigate the effects of extraction time, ethanol concentration, material liquid ratio, and extraction power on the extraction efficiency of flavonoids from *Rosa sterilis*, as well as to provide a reasonable data range for response surface design. The experimental scheme is shown in Table 1.

### 3.2. Optimization Method

#### 3.2.1. Response Surface Methodology

RSM can evaluate the effects of several process variables and their interactions on response variables [39]. 29 groups of experimental conditions were obtained by four factors and three levels optimization experiment. The mathematical model is established using the Design-Expert 8.0.6 software package to acquire the optimum conditions for technological progress. The test factors and levels are shown in Table 2.

#### 3.2.2. ANN Modeling

**Figure 3:**
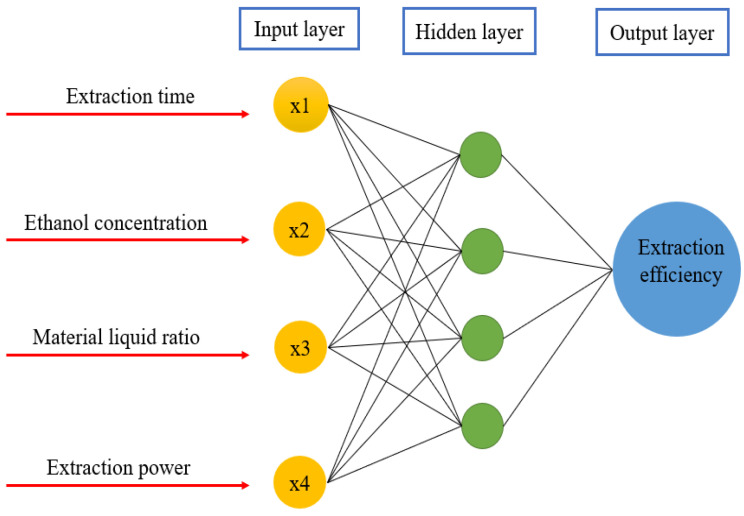
Structure of BP neural network.

BP (Back propagation) is a simple multilayer feed forward neural network based on the error back propagation algorithm [40]. As shown in Figure 3, there is the structure of an input layer, an intermediate layer (hidden layer) and an output layer. BP network can be used to learn and store the relationship between input and output. The weights and thresholds of the network are constantly updated during learning process to obtain the minimum square error of the actual output and model output. When a pair of learning samples is input to the network, the neuron activation function is adjusted from the output layer to the input layer to obtain an input response in the output layer neuron. With the input of training data, the accuracy of the network model is continually improved with the feedback process, and a network model which can best reflect the real relationship is obtained. When the mean squared error (MSE) of the test set reaches the minimum value, the network training is supposed to be completed and the weights are determined.

All input and output values were normalized between 0.1 and 0.9, and the normalized value of input data was described using the following Formula:

(2) $$K=2\times (x_{i}-x_{\min})/(x_{\max}-x_{\min})-1$$

where K was the normalized value of an input data $x_{i}$, $x_{\min}$ and $x_{\max}$ were the minimum and maximum values of variables, respectively.

(3) $$W_{i}=\sum_{i=1}^{n}w_{i}kx_{i}$$

where $W_{i}$ was the connection weight; $x_{i}$ was the value of a neuron in the input layer; $w_{i}k$ is the corresponding connection weight between neuron i in the input layer and neuron j in the hidden layer.

The tangent sigmoid (tansig) function was used between the input and hidden layers, which was described by the following equation:

(4) $$f(x)=2/(1+e^{-2x})-1$$

The linear transfer function (purelin) was used between the hidden and output layers, which is given below:

(5) f(x)=x

The output was produced by the weight and bias of neurons through the activation function:

(6) $$Y=f\times (W_{i}+b)$$

where Y and frepresent the output, activation function, respectively, $W_{i}$ was the connection weight and b was called the bias.

The relative influence of the individual variable was calculated by the following Garson Formula [41]:

(7) $$G_{at}=\frac{\sum_{e}^{n}\left(\frac{\left|w_{ae}\right|}{\sum_{h}^{m}\left|w_{he}\right|}|w_{et}|\right)}{\sum_{z}^{n}(\sum_{l}^{n}\left(\frac{\left|w_{al}\right|}{\sum_{h}^{m}\left|w_{he}\right|}|w_{et}|\right)}$$

where $G_{at}$ was the percentage of influence of the input variable $x_{a}$ on the output variable $y_{t}$; w denoted the connection weight; and a, e, and t represented the number of neurons in the input layer, hidden layer, and output layer, respectively.

#### 3.2.3. Optimization of ANN-GA and ANN-PSO

**Figure 4:**
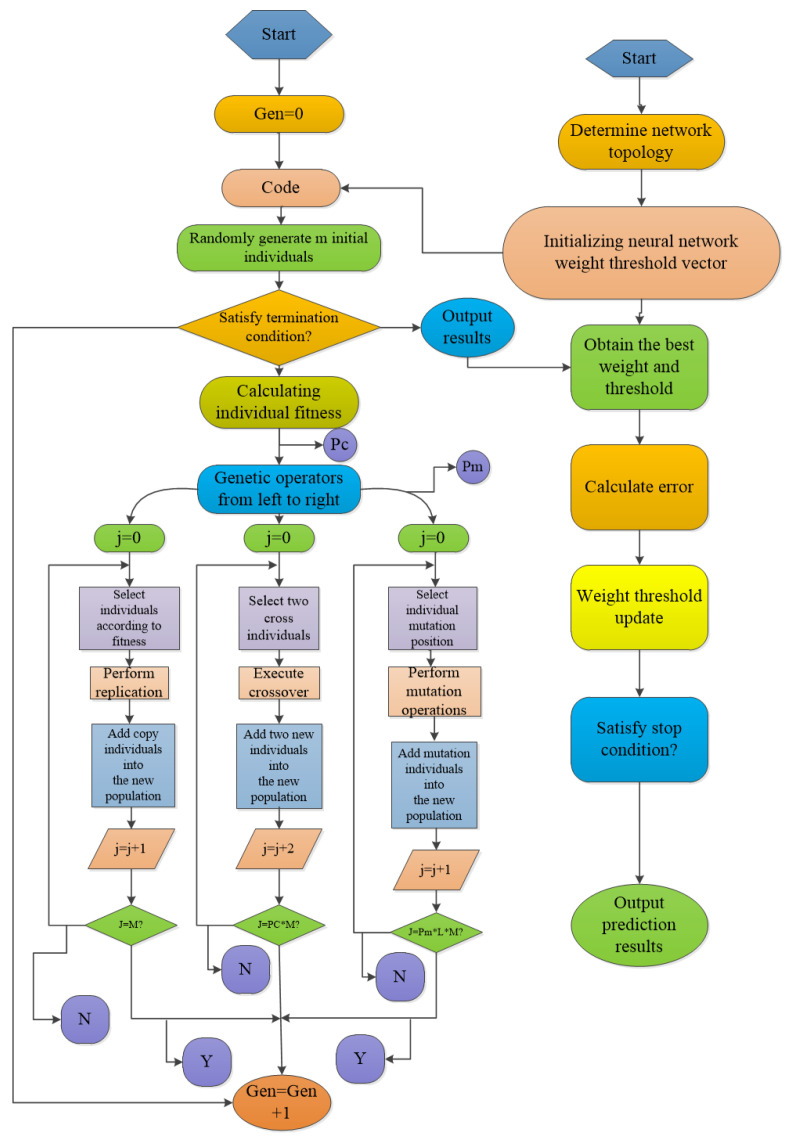
The flow chart of GA optimization process.

ANN-GA and ANN-PSO are modeled and optimized using MATLAB 2016a. GA is a method to search the optimal solution by simulating the natural evolution process. GA analyzes the feasible solution in the self-constraint condition of the function, and it does not depend on the gradient information to find the global optimal solution with large probability. Its global search performance is used to optimize the weights of BP neural network to enhance the generalization ability and prediction accuracy of BP neural network [42]. The flow chart of GA is shown in Figure 4.

**Figure 5:**
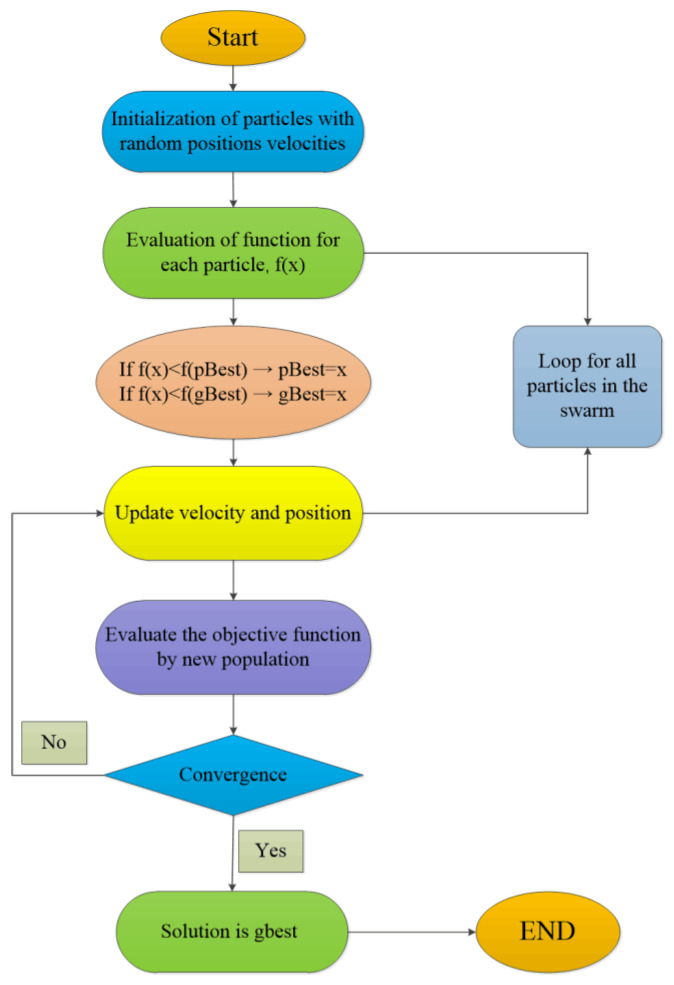
Flow chart of the PSO.

Particle swarm optimization (PSO) algorithm is a search algorithm proposed by Kennedy and Eberhart to simulate the social behavior of birds in the bird swarm. It finds the optimal solution by “flying” individuals called particles in the super dimensional search space. Each particle will adjust its trajectory according to its best position (local optimum) and the best particle (global optimum) in the whole population, which increases the randomness of particles and quickly converges to a reasonable global minimum of the rational solution [43]. The flow chart of PSO is shown in Figure 5.

### 3.3. Importance Ranking

Based on the response surface data, four parameters which affect the extraction efficiency of flavonoids are rearranged using RF, F-test, GBRT, and Garson formula.

Random forest is a model method that integrates mathematics and statistics, it has the advantages of high classification accuracy, resistance to excessive training, effective processing of large data sets, no need for standardized features, and only requires a few parameter optimizations. The GBRT algorithm is based on the boosting algorithm framework. Its basic idea is to construct a GBRT binary classification model based on multiple regression tree sub models, and it continuously learns residuals using the regression tree and reduces the deviation of the whole classification model.

### 3.4. Determination of Free Radical Scavenging Ability

#### 3.4.1. Scavenging Effect of Flavonoids on 2,2-diphenyl-1-picrylhydrazine (DPPH)

First, 7.888 mg DPPH was weighed and contained to 100 mL capacity bottle with 65% ethanol solution to obtain 0.2 mol/L DPPH ethanol solution. An amount of 0.01 g of flavonoids from *Rosa sterilis* was weighed, put into a 100 mL capacity bottle with 60% ethanol solution to obtain a flavonoid solution of 0.1 mg/mL. Then, 0.1 mg/mL flavonoid solution 1, 2, 3, …, 9, 10 mL, respectively, was extracted and put into 10 mL capacity bottles with 60% ethanol solution and mixed. This enables the sample solution to be determined.

An amount of 5 mL of the DPPH ethanol solution was taken, 5 mL of a flavonoid solution of different concentrations was added, and the mixture was shaken well; the light was avoided at room temperature for 30 min, and the absorbance was measured at 517 nm. The experimental control was the absorbance of 5 mL of sample solution and 5 mL of anhydrous ethanol (eliminating the interference of the color of sample solution itself on the experimental determination); the blank control was the absorbance of 5 mL of DPPH solution and 5 mL of anhydrous ethanol; and the sample control was the absorbance of 5 mL of DPPH solution and 5 mL of sample solution. The DPPH radical scavenging efficiency K (%) was calculated by Formula (8):

(8) $$K(\%)=[A_{0}-(A_{1}-A_{2})]/A_{0}\times 100\%$$

where *A*_0_ was the control absorbance of the blank; *A*_1_ was the control absorbance of the sample; *A*_2_ was the experimental control absorbance.

#### 3.4.2. Scavenging Effect of Flavonoids on Superoxide Anion Radical (O^2−^·)

First, 4.5 mL Tris-HCl solution of 0.05 mol/L and pH 8.2 was added into several colorimetric tubes respectively, and the mixture was heated to 25 min in a sewerage bath at 25 °C. Then, 0.1 mL flavonoids extract of different concentrations and 0.3 mL pyrogallol of 3 mmol/L were added. After mixing, the water bath was kept at 20 °C for 6 min, then 2 drops of 8 mol/L HCl were added immediately to terminate the reaction, and the absorbance was measured at 320 nm. In the blank control, distilled water was used to replace the flavonoids extract, and the other steps were the same. In the experimental control, 0.3 mL distilled water was used to substitute pyrogallol, and the remaining steps were the same. The superoxide anion radical scavenging efficiency K (%) was calculated by Formula (9):

(9) $$K(\%)=[B_{0}-(B_{1}-B_{2})]/B_{0}\times 100\%$$

where *B*_0_ was the control absorbance of the blank; *B*_1_ was the control absorbance of the sample; *B*_2_ was the experimental control absorbance.

#### 3.4.3. Scavenging Effect of Flavonoids on Hydroxyl Radical ·OH

First, 1 mL 0.75 mol/L phenanthroline, 2 mL PBS solution with pH value of 7.45, 1 mL distilled water, 1 mL 0.75 mmol/L FeSO_4_ solution, and 1 mL 0.01% H_2_O_2_ were added into the test tube. The mixture is sufficiently blended and the constant temperature water bath 1 h is performed at 37 °C as a blank control. An amount of 1 mL water instead of H_2_O_2_ was used as the experimental control, and 1 mL of sample solution was used instead of 1 mL water as the sample control. The absorbance was measured at 536 nm. The hydroxyl radical scavenging efficiency K (%) was calculated by Formula (10):

(10) $$K\%=(A_{s}-A_{p})/(A_{b}-A_{p})\times 100\%$$

where *A_p_* was the control absorbance of the blank; *A_s_* was the control absorbance of the sample; *A_b_* was the experimental control absorbance.

## 4. Results and Discussion

### 4.1. Single Factor Experiment

**Figure 6:**
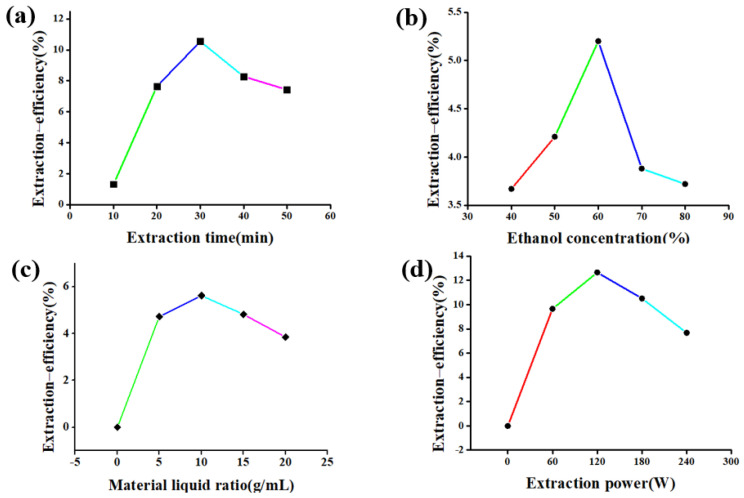
The effect of extraction time (**a**), ethanol concentration (**b**), material liquid ratio (**c**), extraction power (**d**).

The effect of extraction time on the extraction efficiency of flavonoids from *Rosa sterilis* was shown in Figure 6a. With the extension of extraction time, the extraction efficiency of flavonoids first increased and then decreased, and the extraction efficiency reached the maximum at 30 min. The reason for the change of flavonoids extraction efficiency may be that the intracellular flavonoid rapidly increases in the solvent less than 30 min, so the extraction efficiency increases significantly. When it was greater than 30 min, the ultrasonic mechanical effect and thermal energy intensified the molecular motion and the structure of flavonoids was destroyed. At the same time, the extraction of long duration caused the invasion of impurity, and the extraction efficiency of flavonoids decreased.

The effect of ethanol concentration on the extraction efficiency of flavonoids from *Rosa sterilis* is shown in Figure 6b. With the increase of ethanol concentration, the extraction efficiency of flavonoids also increased in a certain concentration range. When the ethanol concentration was 60%, the extraction efficiency of flavonoids reached the maximum value. With the increase of ethanol concentration, the extraction efficiency of flavonoids decreased. The reason may be that when the ethanol concentration is higher than 60%, the solubility of certain alcohol soluble and lipophilic impurities increases, which reduces the solubility of flavonoids in the solvent.

The effect of material liquid ratio on the extraction efficiency of flavonoids from *Rosa sterilis* is shown in Figure 6c. With the increase of material liquid ratio, the extraction efficiency of flavonoids increases gradually. When the material liquid ratio is 1:10, the extraction efficiency of flavonoids reaches the maximum value. The material liquid ratio continues to increase, but the extraction rate of flavonoids decreases. The improvement of the extraction efficiency may be due to the fact that the solute cell is well dispersed in the range of the appropriate material liquid ratio and the heating efficiency is high, which is conducive to cell fragmentation and release of intracellular substances.

The effect of extraction power on the extraction efficiency of flavonoids from *Rosa sterilis* is shown in Figure 6d. When the extraction power was 120 W, the extraction efficiency of flavonoids reached the maximum, which may be that, because of the enhancement of the cell destruction action with the increase of the power, the flavonoids dissolution was promoted and the extraction efficiency was improved. When the power is greater than 120 W, too much power causes local high temperature, which destroys the structure of flavonoids and reduces the extraction efficiency of flavonoids.

### 4.2. Optimization Results

#### 4.2.1. Response Surface Optimization

RSM is composed of mathematical and statistical data, which can be used to define the influence of independent variables on the process alone or in combination [44]. The effects of different process parameters on flavonoids extracted from *Rosa sterilis* were investigated by applying the BBD technique of RSM. Table 2 lists the experimental data and prediction data for extracting flavonoids from *Rosa sterilis*.

The extraction conditions were optimized based on RSM, and the model parameters were verified. The optimal extraction conditions were as follows: extraction time 40 min, ethanol concentration 50.9%, material liquid ratio 1:8.82, extraction power 148.87 W. The maximum predictive value of the model is 11.0688%. The verification experiment is carried out by adjusting the optimization condition (40 min in extraction time, 51% of ethanol concentration, 1:9 of material liquid ratio and extraction power of 150 W). Three repeated experiments showed that the experimental value (extraction efficiency 10.5571%) was consistent with the predicted value, and the absolute error was 0.5117. The good correlation between these results demonstrates the effectiveness of the RSM model, which accurately and reliably reflects the expected optimization effect.

Analysis of variance (ANOVA) is used to detect the significance of the model and the fitting degree of the regression equation. It can obtain the linear and quadratic effects of the treatment variables, their interactions and the coefficients of the response variables. According to the data obtained (Table 3), the *p* value is less than 0.05, indicating that the model is significant. *R*^2^ = 0.9771 describes that there is good compatibility between test results and models of response surfaces. This model is suitable because the F value of the model is 42.61, which indicates that the order of influence of various factors on the flavonoid extraction efficiency of *Rosa sterilis* is C (material liquid ratio) > D (extraction power) > A (extraction time) > B (ethanol concentration) (Table S1). Since its F value is higher, the *p* value is lower, and there is not a significant lack of fit.

In order to describe the relationship between flavonoids extraction efficiency and related parameters, multivariate analysis was carried out, and the second order multiple term fitting regression Formula was (11):

R_1_ = 10.30 + 0.81A + 0.40B − 1.47C + 1.15D − 1.43AB − 1.53AC − 0.048AD + 0.20BC − 1.23BD − 0.59CD − 0.68A^2^ − 1.55B^2^ − 3.50C^2^ − 3.03D^2^ (11)

where R_1_ was the extraction efficiency of flavonoids.

**Figure 7:**
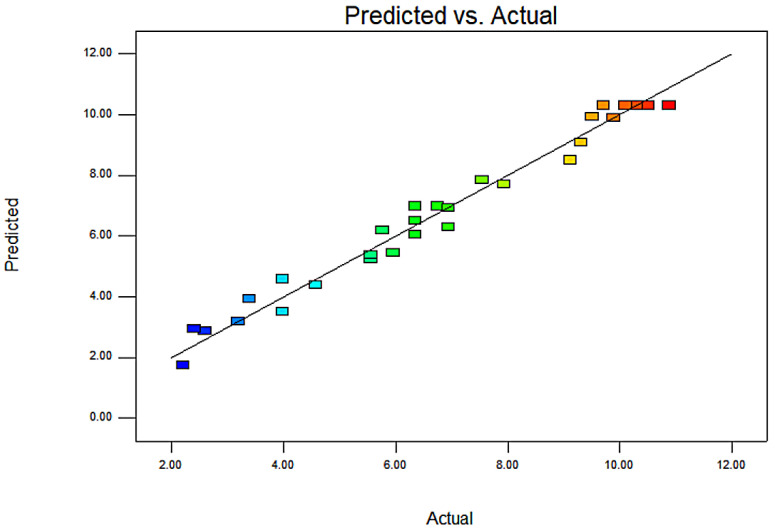
The predicted values versus the actual values.

The relationship between the probability of normal distribution shows that the residual points on the graph are along the straight line, which indicates that the model prediction is accurate (Figure S1). Figure 7 shows that the experimental values of flavonoids extraction efficiency are in good agreement with the predicted values obtained from the model, which indicates that the regression model has high reliability (*R*^2^ = 0.9771).

**Figure 8:**
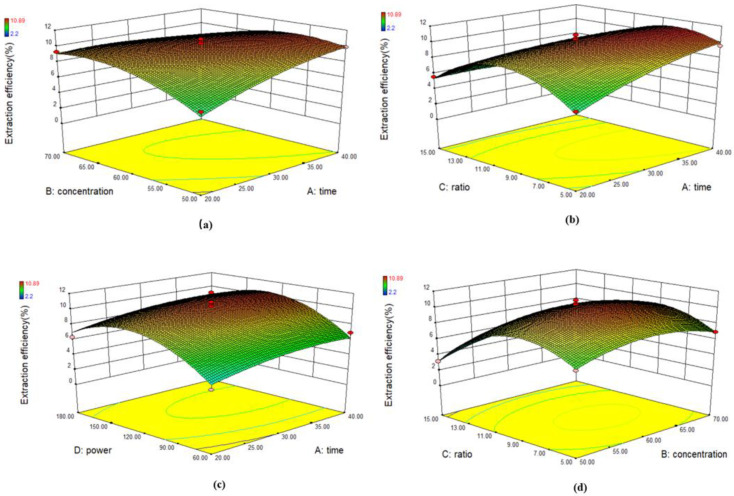

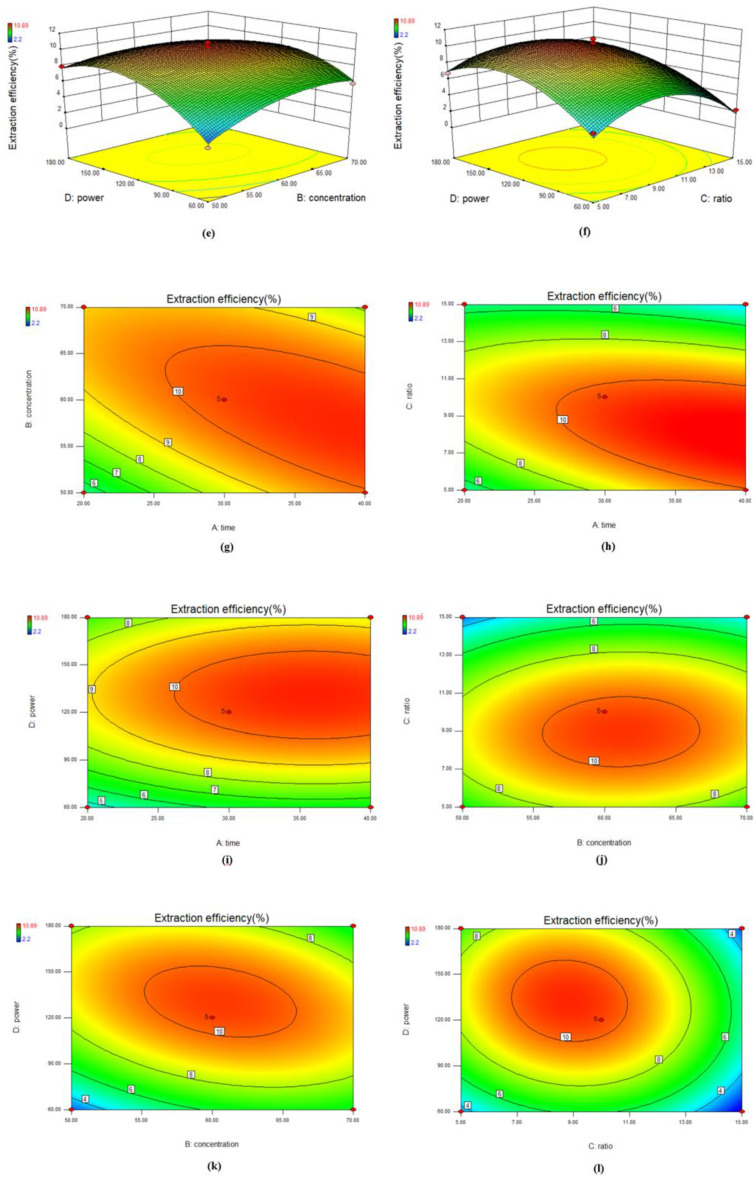
Three-dimensional response surface and 2D contour-lines map plots for the extraction efficiency of flavonoids: (**a**,**g**) Extraction time–Ethanol concentration; (**b**,**h**) Extraction time–Material liquid ratio; (**c**,**i**) Extraction time–Extraction power; (**d**,**j**) Ethanol concentration–Material liquid ratio; (**e**,**k**) Ethanol concentration-Extraction power; (**f**,**l**) Material liquid ratio–Extraction power.

The RSM curve reflects the influence of each variable on the extraction efficiency of flavonoids, and the visualization of the prediction model equation can be obtained by surface response graph. The three-dimensional response surface diagram and two-dimensional contour map of the factors affecting the extraction efficiency of flavonoids are shown in Figure 8. If the slope of the response surface graph is relatively flat, it indicates that the response value has little effect with the change of extraction conditions; on the contrary, if the slope of the response surface is steep, it indicates that the response value has a great influence with the change of extraction conditions. The maximum response value appears in the center of the ellipse and decreases gradually from the center to the edge. The shape of contour map can reflect the degree of interaction; an ellipse shows significant interaction between factors, while a circle shows no significant interaction. There is a significant interaction between the material liquid ratio and the extraction power, while the interaction between other factors is not significant.

#### 4.2.2. ANN-BP Modeling

**Figure 9:**
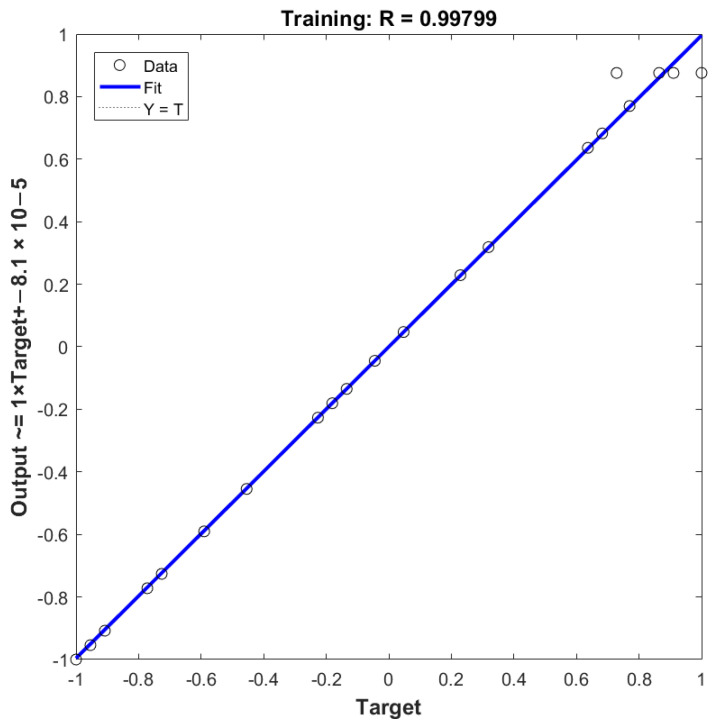
The experimental and predicted data of normalized decontamination.

The experimental data of BP neural network model is based on BBD data. In the whole dataset, 80% of the datas (1–24) are used for training, and 20% of the datas (25–29) are used for testing (Table 2). The *R*^2^ value of the ANN-BP model (0.99799) indicates that the network performance after training is accurate (Figure 9). The hidden layer neuron (n) is determined based on the minimum mean square error (MSE) of the neural network and MSE refers to Figure S2. The influence of each input variable on the output variable is calculated by the Garson formula using weights (Table 4). As the result, it was found that the contribution of the material liquid ratio to the flavonoid extraction efficiency was maximum (45.03%), followed by extraction power (36.43%), ethanol concentration (30.49%) and extraction time (28.57%) (Table S2).

#### 4.2.3. ANN-PSO and ANN-GA

Under the conditions of extraction time of 20.21 min, ethanol concentration of 50%, material liquid ratio of 1:5 and extraction power of 101.89 W, the maximum extraction rate of flavonoids predicted by the ANN-PSO model was 12.7832%, and the corresponding experimental value was 13.2434% (Figure S3). Under the conditions of extraction time of 38.36 min, ethanol concentration of 54.69%, material liquid ratio of 1:7.89 and extraction power of 175.78 W, the maximum extraction efficiency of flavonoids predicted by the ANN-GA model was 13.7512%, and the corresponding experimental value was 14.0137% (Figure S4). The absolute errors of prediction results and experimental results for ANN-PSO and ANN-GA models are 0.4602 and 0.2625, respectively. The results indicated that the artificial neural network genetic algorithm was suitable for predicting the extraction efficiency of flavonoids in *Rosa sterilis* (Table 5).

### 4.3. Importance Ranking

**Figure 10:**
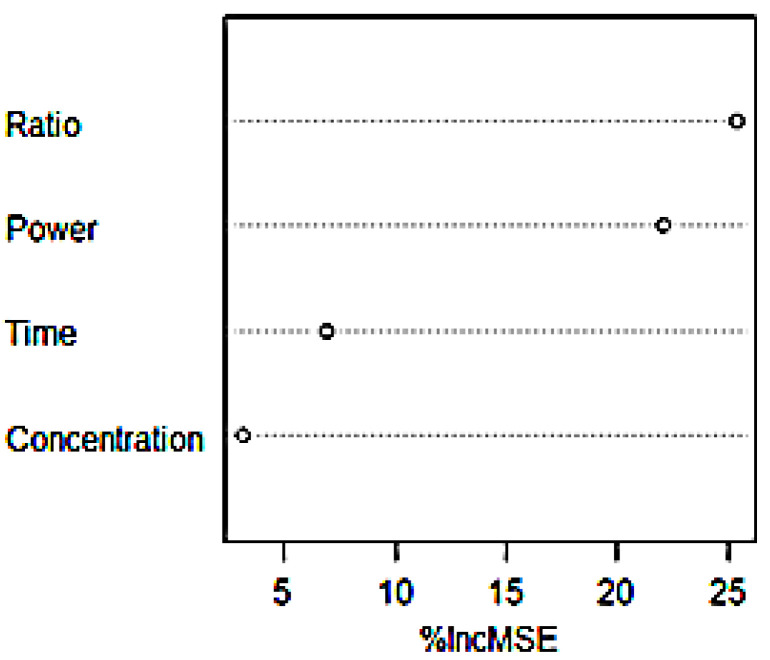
Importance ranking of single factor in RF.

Random forest, F-test, GBRT, and Garson formula were used to rank the four parameters affecting the extraction efficiency of flavonoids. The ranking of parameters by random forest was shown in Figure 10, the ranking of four parameters by GBRT was shown in Figure S5, and the relative importance was shown in Table S3. See Table S4 for the importance of the Garson formula. Table 6 shows that the effects of material liquid ratio and extraction power on flavonoid extraction efficiency are the largest among four influencing factors affecting flavonoids extraction. It is possible to increase the flavonoid extraction efficiency by using appropriate material liquid ratio and extraction power.

### 4.4. Free Radical Scavenging Effect

**Figure 11:**
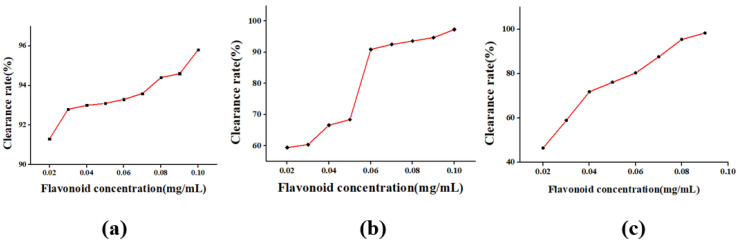
The scavenging efficiency of DPPH (**a**), O^2−^· (**b**), ·OH (**c**).

The antioxidative mechanism of flavonoids, one of the most well studied antioxidants, works by directly capturing and scavenging radicals such as superoxide anion (O^2−^·) and ·OH, hydrogen donor plays a role in free radicals. Blocking the hydrogen deprivation reaction or stopping the free radical chain reaction chain by the reaction that removes hydrogen, the oxygen radical reaction and the lipid peroxidation reaction are prevented or inhibited, and the production of toxic substances such as lipid peroxide (LPO) and its metabolite is suppressed. The scavenging efficiency of flavonoids on DPPH, O^2−^· and ·OH radicals is shown in Figure 11.

As can be seen in the figure, as the flavonoid concentration becomes higher, the scavenging efficiency of DPPH, O^2−^· and ·OH by flavonoids is also higher and higher. The results show that the flavonoids of *Rosa sterilis* have strong antioxidant activity.

### 4.5. Kinetics

The extraction kinetics of total flavonoids from *Rosa sterilis* was analyzed. According to Fick’s first law, the kinetic model of the extraction process of flavonoids was as follows:

(12) $$\ln[C_{\infty }/(C_{\infty }-C)]=kt+\ln[C_{\infty }/(C_{\infty }-C_{0})]$$

where *C*_∞_ was the equilibrium concentration; *C* was the flavonoid concentration at time *t*; *k* was the proportional constant; and *C*_0_ was the initial concentration. The model reflects the relationship between the concentration of flavonoids in *Rosa sterilis* and various factors in the extraction process, such as material liquid ratio, ethanol concentration, extraction time and so on.

**Figure 12:**
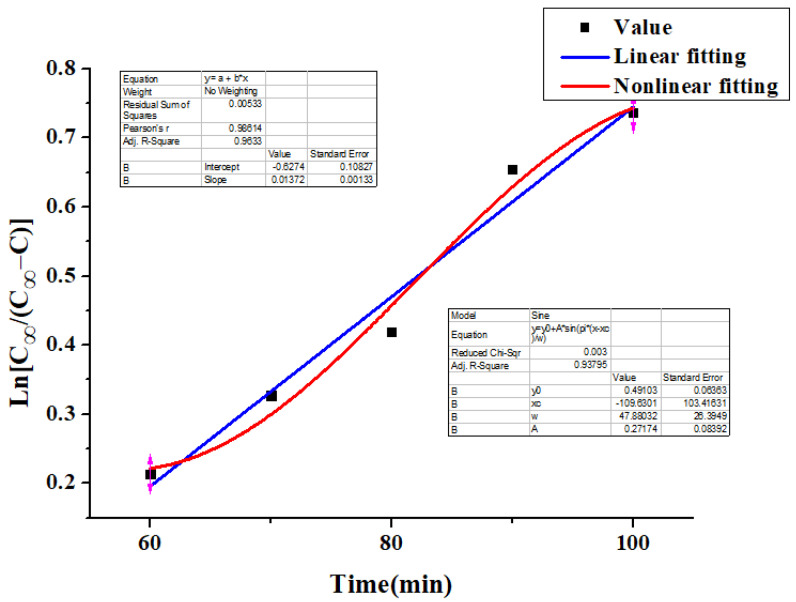
The relationship between ln [*C*_∞_/(*C*_∞_ − *C*)] and time.

Under the condition that the ultrasonic temperature is constant, the extraction efficiency of flavonoids gradually decreases and approaches equilibrium when the extraction time reaches 120 min. Therefore, the flavonoid concentration at 130 min can be used as the equilibrium concentration. Based on the test data in Table S5, the relationship diagram and regression equation between ln [*C*_∞_/ (*C*_∞_ − *C*)] and time *t* are made. The linear fitting and nonlinear fitting are shown in Figure 12.

As a result, there is a good linear relation between ln [*C*_∞_/(*C*_∞_ − *C*)] and extraction time *t*, and the experimental result agrees well with the established kinetic equation, which conforms to the first order kinetic model. It can be seen from Figure 12 that the linear kinetic equation of flavonoids extraction from *Rosa sterilis* is ln [*C*_∞_/(*C*_∞_ − *C*)] = 0.01372 x − 0.6274, *R*^2^ = 0.9633. The nonlinear fitting parameters of dynamics are as follows: y0 is 0.49103, xc is −109.6301, W is 47.88032, A is 0.27174.

## 5. Conclusions

1. The optimum extraction conditions were as follows: extraction time 30 min, ethanol concentration 60%, material liquid ratio 1:10 (g/mL), extraction power 120 W.
2. ANN-BP, ANN-GA and ANN-PSO were used to optimize the extraction conditions. The results show that the ANN-GA algorithm can predict the experimental results well, and the absolute error is 0.2625.
3. GBRT, F-test, Garson formula and random forest were used to analyze the importance of influencing factors. The results show that the material liquid ratio has an important influence on the extraction process.
4. The scavenging effects of flavonoids on DPPH, O^2−^· and ·OH were determined. The results show that flavonoids have strong antioxidant activities, and *Rosa sterilis* is a good source of natural antioxidants.
5. The kinetics of the extraction process were studied, and the linear and nonlinear fitting results show that the extraction process of flavonoids conforms to Fick’s first law.

## Figures and Tables

**Table 1:** Single factor experimental design.

	Extraction Time (min)	Ethanol Concentration (%)	Material Liquid Ratio (g/mL)	Extraction Power (W)
Single factor1	5	60	1:10	120
	10	60	1:10	120
	…	…	…	…
	45	60	1:10	120
	50	60	1:10	120
Single factor2	30	10	1:10	120
	30	20	1:10	120
	…	…	…	…
	30	80	1:10	120
	30	90	1:10	120
Single factor3	30	60	1:2	120
	30	60	1:4	120
	…	…	…	…
	30	60	1:18	120
	30	60	1:20	120
Single factor4	30	60	1:10	10
	30	60	1:10	20
	…	…	…	…
	30	60	1:10	190
	30	60	1:10	200

**Table 2:** Analysis of variance (ANOVA) for response surface quadratic model and BP, RF predicted values.

Run	Extraction Time (min)	Ethanol Concentration (%)	Material Liquid Ratio (g/mL)	Extraction Power (W)	Extraction Efficiency (%)	Predicted Value (%)	BP Predicted Value (%)	RF Predicted Value (%)
1	20	70	10	120	9.31	5.44	9.31	6.40
2	40	60	15	120	3.39	9.9	3.39	6.40
3	30	60	15	180	2.6	9.08	2.6	5.32
4	30	50	15	120	3.19	7.84	3.19	5.36
5	40	60	5	120	9.51	3.49	9.51	7.46
6	30	70	10	180	6.35	1.75	6.35	7.32
7	20	60	10	60	3.98	6.99	3.98	5.47
8	30	50	10	180	7.93	2.87	7.93	6.25
9	20	60	5	120	5.56	4.58	5.56	7.13
10	30	60	10	120	9.71	6.29	10.35	8.67
11	30	50	5	120	6.35	6.98	6.35	6.72
12	20	60	10	180	6.35	8.5	6.35	6.84
13	30	60	5	60	3.98	6.52	3.98	5.58
14	40	50	10	120	9.89	6.92	9.89	6.86
15	20	50	10	120	5.96	3.2	5.96	6.66
16	30	60	15	60	2.2	4.38	2.2	4.48
17	30	60	5	180	6.75	5.25	6.75	7.09
18	30	70	10	60	5.76	9.92	5.76	5.48
19	30	60	10	120	10.3	5.38	10.35	8.73
20	40	70	10	120	7.54	3.93	7.54	7.90
21	30	60	10	120	10.5	2.94	10.35	8.71
22	30	50	10	60	2.4	6.2	2.4	5.88
23	30	60	10	120	10.89	7.72	10.35	8.60
24	30	70	15	120	4.57	6.04	4.57	5.27
25	20	60	15	120	5.56	10.3	4.33	4.70
26	40	60	10	60	6.94	10.3	4.65	6.06
27	40	60	10	180	9.12	10.3	11.73	7.61
28	30	70	5	120	6.94	10.3	10.19	7.26
29	30	60	10	120	10.1	10.3	10.35	8.90

**Table 3:** Analysis of variance (ANOVA) for response surface quadratic model.

Source	Sum of Squares	Degree of Freedom	Mean Square	F-Value	*p*-Value	
Model	196.58	14	14.04	42.61	<0.0001	significant
A-time	7.79	1	7.79	23.64	0.0003	
B-concentration	1.88	1	1.88	5.71	0.0316	
C-ratio	25.75	1	25.75	78.15	<0.0001	
D-power	15.96	1	15.96	48.43	<0.0001	
AB	8.12	1	8.12	24.65	0.0002	
AC	9.36	1	9.36	28.41	0.0001	
AD	9.03E-03	1	9.03E-03	0.027	0.8709	
BC	1.60E-01	1	0.16	0.47	0.5027	
BD	6.1	1	6.1	18.51	0.0007	
CD	1.4	1	1.4	4.26	0.058	
A^2^	3.03	1	3.03	9.19	0.009	
B^2^	15.55	1	15.55	47.18	<0.0001	
C^2^	79.33	1	79.33	240.71	<0.0001	
D^2^	59.44	1	59.44	180.35	<0.0001	
Residual	4.61	14	0.33			
Lack of Fit	3.84	10	0.38	1.98	0.267	not significant
Pure Error	0.78	4	0.19			
Cor Total	201.2	28				

**Table 4:** The weights and biases of BP-ANN in input-hidden layers (w_i_ and b_i_) and hidden-output layers (w_j_ and b_j_).

Number of Neurons	w_i_						
	Input Weights				Input Bias	Layer Weights	Layer Bias
	Extraction Time	Ethanol Concentration	Material Liquid Ratio	Extraction Power			
1	−1.227	1.201	−0.0184	1.803	2.49	0.802	0.46
2	1.174	0.941	−1.143	−1.621	−1.936	0.149	
3	1.569	−0.581	−1.425	1.17	−1.383	0.69	
4	0.097	0.229	2.359	0.757	−0.83	0.477	
5	1.279	−1.365	0.202	1.631	−0.277	0.172	
6	0.374	−1.649	1.824	0.118	0.277	−0.507	
7	−1.595	−1.68	0.908	−0.095	−0.83	0.333	
8	−1.594	0.949	0.44	1.601	−1.383	−0.833	
9	−0.543	−0.028	1.839	−1.587	−1.936	0.252	
10	1.127	−1.406	1.718	−0.009	2.49	0.322	

**Table 5:** Comparison between the predicted extraction efficiency of flavonoids by using BBD, ANN-GA and ANN-PSO models and the experimental results.

Models		Independent Parameters			Experiment (%)	Prediction (%)	Absolute Error (%)
	A	B	C	D			
BBD	40	50.9	8.82	148.87	10.5571	11.0688	0.5117
ANN-GA	38.36	54.69	7.89	175.78	14.0137	13.7512	0.2625
ANN-PSO	20.21	50	5	101.89	13.2434	12.7832	0.4602

**Table 6:** Importance ranking of single factor in F-test, RF, GBRT, and Garson formula.

Methods	Extraction Time (min)	Ethanol Concentration (%)	Material Liquid Ratio (g/mL)	Extraction Power (W)
F-test	3	4	1	2
RF	3	4	1	2
GBRT	3	4	1	2
Garson-formula	4	3	1	2

## Data Availability

Data is contained within the article.

## Supplementary Materials

The following are available online, Figure S1: The normal probabilities versus internally studentized residuals, Figure S2: Mean square error (MSE) of neurons in the BP-ANN model, Figure S3: Extraction efficiency versus iteration, Figure S4: Decontamination efficiency versus generation, Figure S5: Importance ranking of single factor in GBRT, Table S1: Relative influence of input variables, Table S2: Relative influence of variables, Table S3: The relative importance of GBRT, Table S4: The relative importance of Garson formula, Table S5: Extraction efficiency of flavonoids under different extraction time.

## Sample Availability

Samples of the compounds are not available from the authors.

## References

[1] Li J.L., Quan W.X., Li C.C. (2018). Effects of ecological factors on content of flavonoids in *Rosa sterilis* from different karst areas of Guizhou, SW China. Pak. J. Bot..

[2] Panche A.N., Diwan A.D., Chandra S.R. (2016). Flavonoids: An overview. J. Nutr. Sci. Vitaminol..

[3] Liu M.H., Zhang Q., Zhang Y.H. (2016). Chemical Analysis of Dietary Constituents in *Rosa roxburghii* and *Rosa sterilis* Fruits. Molecules.

[4] He J.Y., Zhang Y.H., Ma N., Zhang X.L., Liu M.H., Fu W.M. (2016). Comparative analysis of multiple ingredients in *Rosa roxburghii*, and *R. sterilis*, fruits and their antioxidant activities. J. Funct. Foods.

[5] Wang L., Lv M.J., An J.Y. (2021). Botanical characteristics, phytochemistry and related biological activities of Rosa roxburghii Tratt fruit and the potential use in functional foods: A review. Food Funct..

[6] Oniszczuk A., Widelska G., Jtowicz A.W. (2019). Content of phenolic compounds and antioxidant activity of new Gluten-Free pasta with the addition of chestnut flour. Molecules.

[7] Schneider J.R., Müller M., Klein V.A. (2020). Soybean plant metabolism under water deficit and xenobiotic and antioxidant agent application. Biology.

[8] Guilherme R., Aires A., Rodrigues N. (2020). Phenolics and antioxidant activity of green and red sweet peppers from organic and conventional agriculture: A comparative study. Agriculture.

[9] Park C.H., Min S.Y., Yu H.W. (2020). Effects of apigenin on RBL-2H3, RAW264.7, and HaCaT Cells: Anti-Allergic, Anti-Inflammatory, and Skin-Protective activities. Int. J. Mol. Sci..

[10] Wen R., Lv H., Jiang Y. (2018). Anti-inflammatory flavone and chalcone derivatives from the roots of *Pongamia pinnata* (L.) Pierre. Phytochemistry.

[11] Li X., Jiang X., Sun J. (2017). Cytoprotective effects of dietary flavonoids against cadmium-induced toxicity. Ann. N. Y. Acad. Sci..

[12] Freitas C.D., Rocha M., Sacramento C.Q. (2020). Agathisflavone, a biflavonoid from *Anacardium occidentale* L. inhibits influenza virus neuraminidase. Curr. Top. Med. Chem..

[13] Seleem D., Pardi V., Murata R.M. (2017). Review of flavonoids: A diverse group of natural compounds with anti-Candida albicans activity in vitro. Arch. Oral Biol..

[14] Javier E., Julia O., Leonora M. (2017). Structure-activity and lipophilicity relationships of selected antibacterial natural flavones and flavanones of chilean flora. Molecules.

[15] Kim S.Y., Wie G.A., Cho Y.A. (2017). The role of red meat and flavonoid consumption on cancer prevention: The Korean cancer screening examination cohort. Nutrients.

[16] Sun G.W., Qiu Z.D., Wang W.N. (2016). Flavonoids extraction from propolis attenuates pathological cardiac hypertrophy through PI3K/AKT signaling pathway. Evid. Based Complementary Altern. Med..

[17] Oniszczuk A., Jtowicz A.W., Oniszczuk T. (2020). Opuntia fruits as food enriching ingredient, the first step towards new functional food products. Molecules.

[18] At A., Img A., Kpp B. (2021). Inhibition of calcitriol inactivating enzyme CYP24A1 gene expression by flavonoids in hepatocellular carcinoma cells under normoxia and hypoxia. Arch. Biochem. Biophys..

[19] Masad R.J., Haneefa S.M., Mohamed Y.A. (2021). The immunomodulatory effects of honey and associated flavonoids in cancer. Nutrients.

[20] Zhu L., Chen J., Tan J. (2017). Flavonoids from *Agrimonia pilosa* Ledeb: Free radical scavenging and DNA oxidative damage protection activities and analysis of bioactivity-structure relationship based on molecular and electronic structures. Molecules.

[21] Kai N. (2012). Optimization of total flavonoids extraction from mulberry leaf using an ethanol-based solvent system. J. Med. Plants Res..

[22] Kong F., Yu S., Bi Y. (2016). Optimization of process parameters and kinetic model of enzymatic extraction of polyphenols from Lonicerae Flos. Pharmacogn. Mag..

[23] Li X., Liu Y., Di D. (2016). A formaldehyde carbonyl groups-modified self-crosslinked polystyrene resin: Synthesis, adsorption and separation properties. Colloid. Surface A..

[24] He B.H., He J., Wang G.X. (2016). Photoinduced controlled/”living” polymerization of methyl methacrylate with flavone as photoinitiator. J. Appl. Polym. Sci..

[25] Sun T., Chen S., Huang H. (2017). Metabolic profile study of 7, 8-dihydroxyflavone in monkey plasma using high performance liquid chromatography–tandem mass spectrometry. J. Chromatogr. B.

[26] Bo N., Chen L., Yi C. (2019). A high performance liquid chromatography method for simultaneous detection of 20 bioactive components in tea extracts. Electrophoresis.

[27] Ouédraogo J.C.W., Dicko C., Kini F.B. (2018). Enhanced extraction of flavonoids from *Odontonema strictum* leaves with antioxidant activity using supercritical carbon dioxide fluid combined with ethanol. J. Supercrit. Fluid.

[28] Oroian M., Dranca F., Ursachi F. (2019). Comparative evaluation of maceration, microwave and ultrasonic-assisted extraction of phenolic compounds from propolis. Int. J. Food Sci. Technol..

[29] Li D., Qian Y., Tian Y.J. (2017). Optimization of ionic liquid-assisted extraction of biflavonoids from *Selaginella doederleinii* and evaluation of its antioxidant and antitumor activity. Molecules.

[30] Zhang H., Xie G., Tian M. (2016). Optimization of the ultrasonic-assisted extraction of bioactive flavonoids from *Ampelopsis grossedentata* and subsequent separation and purification of two flavonoid aglycones by high-speed counter-current chromatography. Molecules.

[31] Hao K., Hu W., Hou M. (2019). Optimization of ultrasonic-assisted extraction of total phenolics from *Citrus aurantium* L. blossoms and evaluation of free radical scavenging, Anti-HMG-CoA reductase activities. Molecules.

[32] Zheleva-Dimitrova D., Zengin G., Ibrahime S.K. (2020). Identification of bioactive compounds from *Rhaponticoides iconiensis* extracts and their bioactivities: An endemic plant to Turkey flora. J. Pharmaceut Biomed..

[33] Reddy A.V.B., Moniruzzaman M., Madhavi V., Jaafar J. (2020). Recent improvements in the extraction, cleanup and quantification of bioactive flavonoids. Stud. Nat. Prod. Chem..

[34] Jia W.W., Chen Z.B., Zhao Y.Y., Li K., Tichnell B., Tang Z.H., Ruso J.M., Liu Z. (2019). The study of ultrasound-assisted extraction of flavonoids from *Polygonum cuspidatum* Sieb. et Zucc. J. Mater. Res..

[35] Ueyama H., Kato Y., Akazawa Y. (2021). Application of artificial intelligence using a convolutional neural network for diagnosis of early gastric cancer based on magnifying endoscopy with narrow-band imaging. J. Gastroenterol. Hepatol..

[36] Taddeo M., Floridi L. (2018). Regulate artificial intelligence to avert cyber arms race. Nature.

[37] Hassabis D., Kumaran D., Summerfield C. (2017). Neuroscience-Inspired artificial intelligence. Neuron.

[38] Danna H., Zhou X.L., Si J.Z., Gong X.M., Wang S. (2016). Studies on cellulase-ultrasonic assisted extraction technology for flavonoids from Illicium verum residues. Chem Cent. J..

[39] Mohammadi R., Mohammadifar M.A., Mortazavian A.M., Rouhi M., Ghasemi J.B., Delshadian Z. (2016). Extraction optimization of pepsin-soluble collagen from eggshell membrane by response surface methodology (RSM). Food Chem..

[40] Hu J., Qian Z. (2018). The prediction of adhesive failure between aggregates and asphalt mastic based on aggregate features. Constr. Build. Mater..

[41] Fan M.Y., Hu J.W., Cao R.S., Xiong K.N., Wei X.H. (2017). Modeling and prediction of copper removal from aqueous solutions by nZVI/rGO magnetic nanocomposites using ANN-GA and ANN-PSO. Sci. Rep..

[42] Wang J., Shi P., Jiang P. (2017). Application of BP neural network algorithm in traditional hydrological model for flood forecasting. Water.

[43] Gong Y.J., Li J.J., Zhou Y. (2017). Genetic Learning Particle Swarm Optimization. IEEE Trans. Cybern..

[44] Hammi K.M., Hammami M., Rihouey C. (2016). Optimization extraction of polysaccharide from Tunisian Zizyphus lotus fruit by response surface methodology: Composition and antioxidant activity. Food Chem..

