# Supplementary material for: Artificial Intelligence Assisted Ultrasonic Extraction of Total Flavonoids from Rosa sterilis
Source: Molecules. 2021 Jun 23;26(13):3835. doi: 10.3390/molecules26133835 (PMC8270336; doi:10.3390/molecules26133835)
Supplement: Supplementary file 1 [file molecules-26-03835-s001.zip › molecules-1250742-supplementary.pdf]

# Supplementary material

## Artificial intelligence assisted ultrasonic extraction of total flavonoids from *Rosa sterilis*

**Jing Liu [1,2], Chaochan Li [2,*], Guijie Ding [3,*] and Wenxuan Quan [2]**

1 Key Laboratory of Plant Physiology and Developmental Regulation of Guizhou Province, College of Life Sciences, Guizhou Normal University, Guiyang 550025, China. L2876395771@163.com
2 Guizhou Provincial Key Laboratory for Information Systems of Mountainous Areas and Protection of Ecological Environment, Guizhou Normal University, Guiyang 550001, China. chaochanl@gznu.edu.cn; wenxuanq@gznu.edu.cn
3 Institute for Forest Resources and Environment of Guizhou, Key Laboratory of Forest Cultivation in Plateau Mountain of Guizhou Province, Guizhou University, Guiyang 550025, China. gjdinggzu@126.com
* Correspondence: chaochanl@gznu.edu.cn; gjdinggzu@126.com

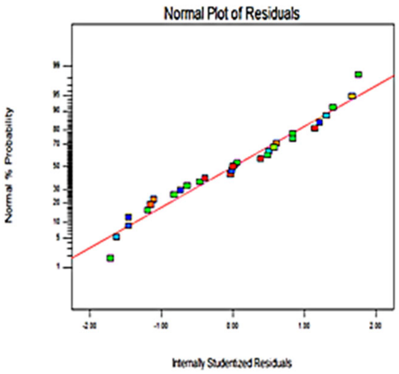


**Figure S1.** The normal probabilities versus internally studentized residuals.

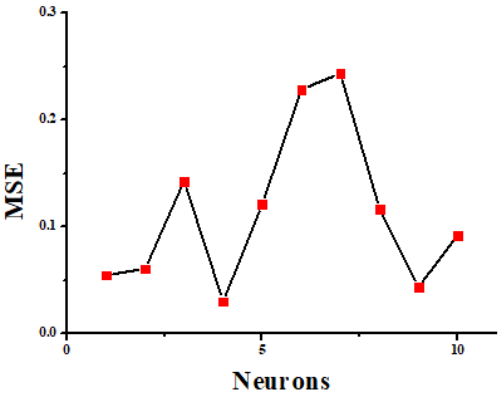


**Figure S2.** Mean square error (MSE) of neurons in the BP-ANN model.

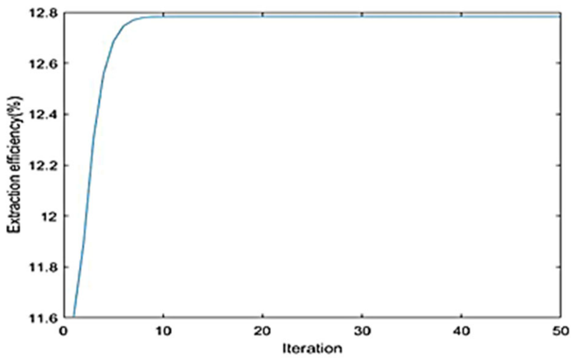


**Figure S3.** Extraction efficiency versus iteration.

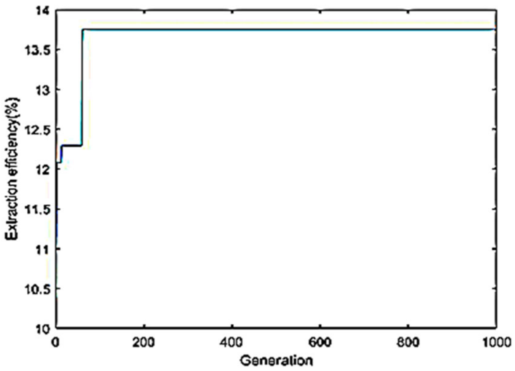


**Figure S4.** Decontamination efficiency versus generation.

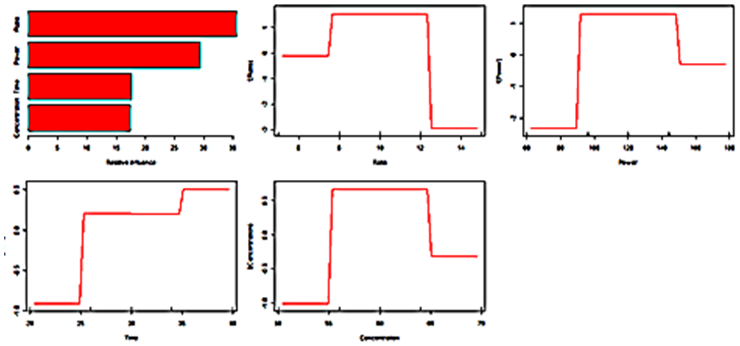

**Figure S5.** Importance ranking of single factor in GBRT.

**Table S1.** Relative influence of input variables.

| Variables | Relative Significance (%) | Order |
|---|---|---|
| Extraction time | 23.64% | 3 |
| Ethanol concentration | 5.71% | 4 |
| Material liquid ratio | 78.15% | 1 |
| Extraction power | 48.43% | 2 |

**Table S2.** Relative influence of variables.

| Variables | Relative Significance (%) | Order |
|---|---|---|
| Extraction time | 28.57% | 4 |
| Ethanol concentration | 30.49% | 3 |
| Material liquid ratio | 45.03% | 1 |
| Extraction power | 36.43% | 2 |

**Table S3.** The relative importance of GBRT.

| Input variables | Relative significance (%) | Order |
|---|---|---|
| Extraction time | 17.65561% | 3 |
| Ethanol concentration | 17.39120% | 4 |
| Material liquid ratio | 35.58806% | 1 |
| Extraction power | 29.36513% | 2 |

**Table S4.** The relative importance of Garson formula.

| Input variables | Relative significance (%) | Order |
|---|---|---|
| Extraction time | 12.99% | 3 |
| Ethanol concentration | 20.89% | 4 |
| Material liquid ratio | 31.53% | 2 |
| Extraction power | 34.60% | 1 |

**Table S5.** Extraction efficiency of flavonoids under different extraction time.

| Extraction time (min) | $\ln[C_\infty/(C_\infty - C)$ |
|---|---|
| 60 | 0.214 |
| 70 | 0.327 |
| 80 | 0.419 |
| 90 | 0.655 |
| 100 | 0.736 |
